# Effect of different ergonomic supports on muscle activity of dentists during posterior composite restoration

**DOI:** 10.7717/peerj.8028

**Published:** 2019-11-07

**Authors:** Manuel López-Nicolás, José A. García-Vidal, Francesc Medina-Mirapeix, Joaquín P. Sánchez-Onteniente, Juan D. Berná Mestre, Rodrigo Martín-San Agustín, María P. Escolar-Reina

**Affiliations:** 1 Department of Dermatology, Stomatology, Radiology and Physical Medicine, University of Murcia, Murcia, Spain; 2 Department of Physiotherapy, University of Murcia, Murcia, Spain; 3 Department of Ophthalmology, Optometry, Otolaryngology and Pathological Anatomy, University of Murcia, Murcia, Spain; 4 Department of Radiology, Virgen de la Arrixaca University Clinical Hospital, El Palmar, Spain; 5 Department of Physiotherapy, University of Valencia, Valencia, Spain

**Keywords:** Musculoskeletal disorders, Ergonomics, Electromyography, Physical therapy modalities, Dentists

## Abstract

**Background:**

The aim of this study was to evaluate the effect of different ergonomic supports on the muscle activity of two trunk muscles while a group of dentists performed a common dental procedure on a phantom head, divided into three tasks.

**Methods:**

A one-way repeated measures study (ANOVA) was conducted on a group of 36 dentists. The middle trapezius and lumbar erector spinae muscles were measured with and without the use of different ergonomic supports (ergonomic stool, magnification lenses and both) using a portable surface electromyography (sEMG) device. Intraclass correlation coefficients (ICCs) and the absolute standard error of measurement (SEM) were used to establish the reliability of the baseline measures without ergonomic supports.

**Results:**

The sEMG showed excellent ICCs (ranging from 0.92 to 0.99) and SEM. Bonferroni post hoc tests showed differences between the three ergonomic supports (*p* < 0.001) in both muscles studied. The lowest muscle activity measurement occurred with the isolated used of magnification lenses. The use of the ergonomic stool increased the muscle activity of the middle trapezius and lumbar erector spinae muscles to a greater extent than the magnification lenses. The combination of the ergonomic stool and the magnification lenses produced a different effect on each muscle.

## Introduction

Back pain is considered one of the most common musculoskeletal disorders (MSDs) among dentists (Finsen, Christensen & Bakke, 1998). Recent studies show that 80% of the general population and over 60% of dentists will suffer back pain during their professional life (Hayes, Cockrell & Smith, 2009; Dajpratham et al., 2010; De Bruyne et al., 2016). According to these authors, higher levels of muscle activity, a strained posture and repetitive movements are the most recognized risk factors for developing MSDs (Biswas et al., 2012; Tran et al., 2016).

In the field of dentistry, a number of strategies have been recommended to reduce the incidence of musculoskeletal pain, such as reducing work time and fatigue (Thakar et al., 2015), limiting the workspace (Custódio, Silva & Brandão, 2012), alternating standing with sitting time (Pîrvu et al., 2014), working with dental assistants (Gosavi, Gosavi & Jawade, 2012), increasing physical activity and stretching (Gosavi, Gosavi & Jawade, 2012; Kumar et al., 2014). Most recently, different ergonomic supports, such as the ergonomic stool and magnification lenses seem to be the most common solutions used by dentists (Branson et al., 2004; Valachi, 2009; Haddad et al., 2012; Tran et al., 2016; De Sio et al., 2018).

Multiple outcome measures have been used to date to demonstrate the utility of the ergonomic stool (Haddad et al., 2012; Tran et al., 2016; De Bruyne et al., 2016) and magnification lenses (Branson et al., 2004; Hayes et al., 2014, 2016; Dable et al., 2014; Rafie et al., 2015), such as pain assessments and functional scales (Hayes et al., 2016), perceived fatigue (Rolander et al., 2005; Ranavolo et al., 2018), motion analysis (Branson et al., 2004; Silva et al., 2016), postural assessment (Branson et al., 2004; Haddad et al., 2012; Dable et al., 2014) and muscle activity (Gandavadi, 2008; Tran et al., 2016; De Bruyne et al., 2016). Surface electromyography (sEMG) is the most extensively used device to describe muscle activity in daily dental work (Rolander et al., 2005; Blanc, Farre & Hamel, 2014). Despite this, very few studies have used sEMG as an outcome measure to analyze the effect of ergonomic supports such as the ergonomic stool on back muscles (Haddad et al., 2012; Tran et al., 2016; De Bruyne et al., 2016), and, according to our research, no studies have focused specifically on the effect of magnification lenses. Consequently, to our knowledge, there are no studies that compare the effect of magnification lenses and ergonomic stool, separately, or combined in muscle activation patterns in the middle and low back muscles. In the present study, we evaluated the influence of different ergonomic supports (ergonomic stool and magnification lenses) on the muscle sEMG activity of two trunk muscles during a posterior composite restoration procedure.

## Materials and Methods

### Study design

We used a one-way repeated measures design. Using a portable sEMG recorder, we measured the muscle activity of two trunk muscles: the medium trapezius (MT) from the dominant upper extremity and the lumbar erector spinae (LES). This procedure was carried out while participants performed three tasks of a posterior composite restoration procedure on a phantom head: tooth drilling, tooth filling and tooth polishing. All participants performed these tasks, first without ergonomic support (baseline measures), and then using three different ergonomic supports with 15 min of rest time between each task. The order in which these different ergonomic supports were used was randomized. Similarly, the order in which each of the tasks were executed was also randomized. In order to examine the reliability of sEMG measures during each task, we also used a test–retest design for the baseline condition using a subsample of participants who completed two sessions, 30 min apart.

### Participants

Thirty-six dentists were recruited (*n* = 36) among post-graduate students and professors of the University of Murcia. Eligible participants were aged 60 years old or less and without self-reported health MSDs in the back, neck and upper limbs. The exclusion criteria were visual acuity problems, previous surgery, medication that may affect the eyes or an inability to wear glasses/prisms. All participants gave written informed consent. This study obtained approval from the Ethics Committee of the University of Murcia (approval No. 986/2014).

### Instrumentation

All dental tasks (and associated sEMG measures) were performed on a simulated dental Workstation comprised of a dental chair with adjustable height and an operating lamp Kavo *Primus 2058*^®^ (Kavo Dental GmbH, Biberach, Germany), a phantom head and dental model KavoG-50 Jaw simulator^®^ (Kavo Dental GmbH, Biberach, Germany) and a conventional stool. Dental materials (e.g., composite) and the dental tools were provided by the researchers. The ergonomic supports used to generate the different conditions were an ergonomic stool with adjustable lumbar support MO Sit Up Balance Op Chair^®^ (MeridentOptergo AB, Mölnlycke, Sweden) and magnification lenses MO Ultralight Flip-up Prism solutions^®^ (MeridentOptergo AB, Mölnlycke, Sweden). These lenses are a novel telemicroscope that provides an increase of 2.5, while the inclination of the lens and the frame provides a correct angle of vision without the need to tilt the head.

Electrical muscle activity of the MT and LES were recorded during the dental tasks using sEMG. A laboratory system, the data-LINK900 software v.7.5 (Biometrics Ltd, Newport, UK), was used. Three active SX230 sEMG sensors (Biometrics Ltd, Newport, UK) were employed for the measurements. sEMG bipolar electrodes were placed on the appropriate locations, using double-sided die-cut tapes provided by the manufacturer. All sEMG signals were amplified and sampled at 1,000 Hz. Raw bipolar Electromyography (EMG) data were processed by using the root-mean-square with 50 ms and 64 Hz filter.

### Procedures

Each participant was evaluated on two separate days, 1 week apart. On the first day, demographic and anthropometric characteristics were obtained by self-report. Additionally, the participants received a talk on ergonomics and the use of magnification lenses and the ergonomic stool. Finally, a professional optician adapted the prismatic glasses to individually fit each dentist, so that participants could become accustomed to using these during the week prior to the data collection of muscle activity.

**Figure 1:**
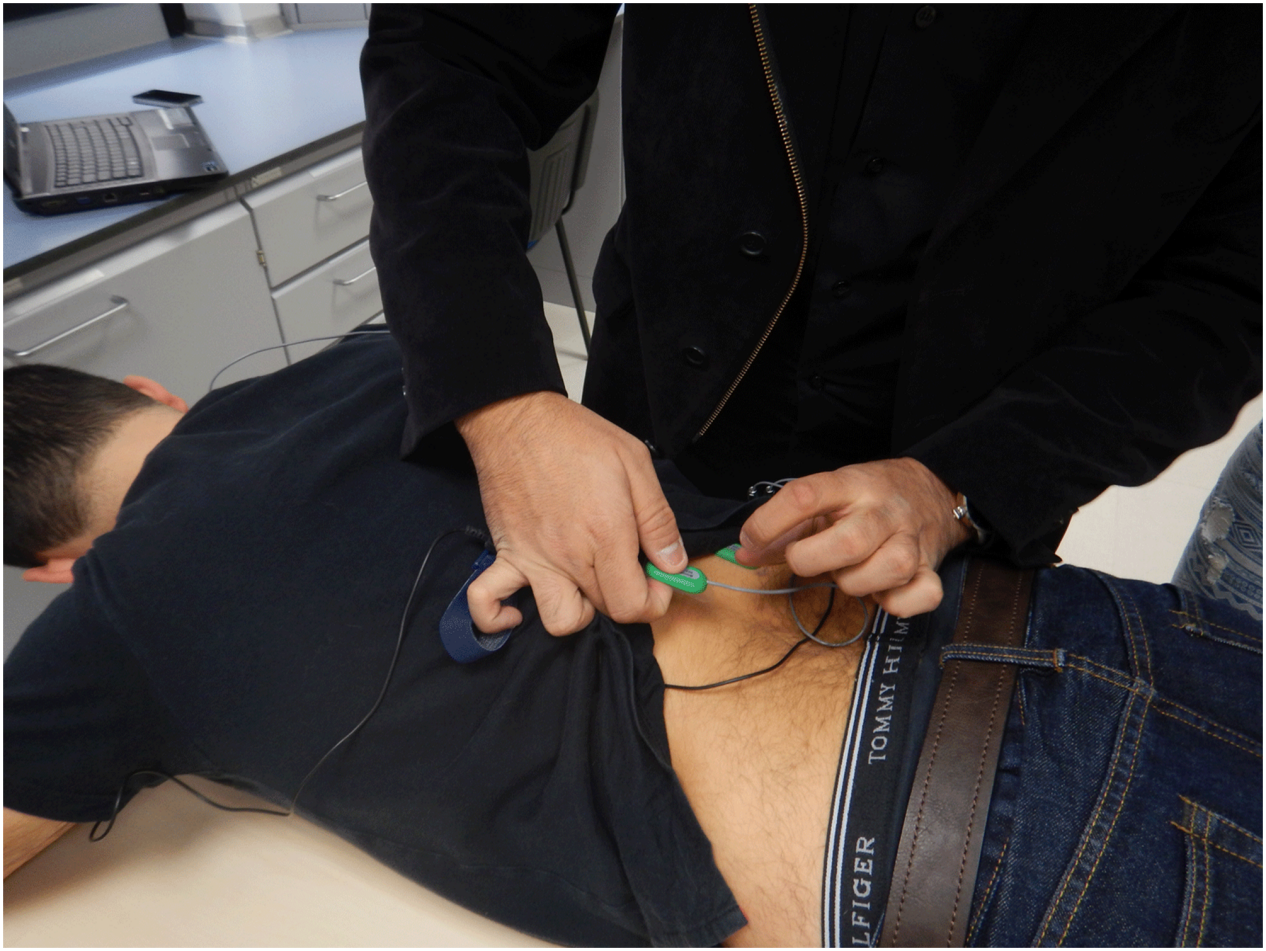
Electrode placement for Lumbar Erector Spinae.

On the second day, data collection was divided into three phases: preparation, baseline recording and experimental records. First, subjects were led to a simulated dental workstation for the application of the sEMG electrodes. The skin over the muscles was cleaned with alcohol. SX230 sensors were placed parallel to the fibers of the selected muscles with double-sided adhesive tape according to the anatomical locations recommended by SENIAM and Motmans, Tomlow & Vissers (2006) (Fig. 1). A ground electrode was placed on the wrist.

To normalize the data obtained, three maximum voluntary isometric contractions (MVICs) of each measured muscle were recorded following a muscle testing procedure. The maximum and mean amplitudes of the sEMG obtained during the functional exercises was then normalized with the highest value of the MVIC and expressed as a percentage of MVIC (% MVIC). This procedure requires three maximal resisted voluntary contractions, performed in the sitting position and in the following order: scapular retraction for MT and lumbar extension for LES. After the collection of the maximum contractions, the participants were allowed to rest for 10 min before commencing the baseline recording.

Prior to recording the measurements, the starting position on the workstation was standardized as follows: the dentist sat next to the doll in a manner that enabled an approach via their dominant side, the feet were separated and placed on the floor, the knees were flexed at 90°, the angle between the lumbar spine and the femur was 110° to avoid rectification of lordosis, the back was straight, the shoulders were parallel to the horizontal plane with the arms and elbows attached to the body. Although all dentists started from the same starting position, they were allowed to adopt their usual posture once the measurement began.

The sEMG baseline recordings were established for each subject while performing the tasks in normal conditions with a conventional stool. The experimental records were established for each of the three different ergonomic conditions (ergonomic stool, magnification lenses and both). Both baseline and experimental EMG measures were averaged for each task, condition and muscle group, and then normalized by their respective MVIC.

### Statistical analysis

IBM SPSS^®^ statistics v.23.0 (IBM, Chicago, IL, USA) was used for statistical analysis. The relative test–retest reliability was evaluated using intraclass correlation coefficients (ICCs), and the absolute reliability was measured using standard error of measurement (SEM). The SEM was presented in absolute reliability terms and as a SEM% by dividing the SEM with the average of the test and retest values. A repeated measures analysis of variance (ANOVA) was used to determine the mean differences in sEMG values for each muscle group between the four ergonomic conditions (conventional stool, ergonomic stool, magnification lenses, ergonomic stool and magnification lenses). In addition, we performed multiple post hoc comparisons tests between the ergonomic conditions to determine which conditions were significantly different from others. For this, we used the Bonferroni *t*-test. We used a significance level of α < 0.05 for all analyses.

Sample size calculations were based on one group being measured across four observations. Using an alpha of 0.05, a power of 0.80 and an estimated moderate treatment effect of 0.03 (partial eta-squared), the necessary sample size (*n*) was estimated to be a minimum of 36 participants, in order to detect a significant treatment effect.

## Results

A total of 36 participants completed all of the tasks with all the ergonomic supports. Their ages ranged from 20 to 59 (39.5 ± 19.5) years old. Of these, 26 (72.2%) participants were included in the test–retest reliability study. In this reliability study, participants and non-participants did not differ with regards to sex (*p* = 0.178 and *p* = 0.425, respectively), age (*p* = 0.953; *p* = 0.310) and education level (*p* = 0.365; *p* = 0.067).

The reliability of the sEMG measurements for each muscle and task is presented in Table 1. All measurements showed excellent ICCs, and all their confidence intervals had a lower limit that was higher than 0.6. The SEM was under 10%. All muscles showed a comparable measurement error between the different tasks.

**Table 1:** Reliability of sEMG measurements for each muscle and task.

Muscle	Task	ICC (95% CI)	SEM	%SEM
MT	TD	0.994 [0.987–0.997]	0.04	3.3
	TF	0.962 [0.917–0.983]	0.05	8.8
	TP	0.999 [0.998–1.000]	0.003	1.3
LES	TD	0.999 [0.998–1.000]	0.002	1.3
	TF	0.992 [0.984–0.996]	0.06	4.3
	TP	0.997 [0.994–0.998]	0.02	2.4

**Note:**

MT, medium trapezius; LES, lumbar erector spinae; TD, tooth drilling; TF, tooth filling; TP, tooth polishing; ICC, intraclass correlation coefficient; SEM, standard error of measurement.

Table 2 shows the mean sEMG values (and SD) expressed as a percentage of MVIC for each of four ergonomic conditions and the three tasks. The results of the one-way repeated measures ANOVA showed that the muscle activity of MT differed significantly across the four ergonomic conditions in the three tasks (*F* = 46.77 *(p* < 0.001), *F* = 40.14 *(p* < 0.001), *F* = 49.83 *(p* < 0.001), respectively). The Bonferroni post hoc tests showed that the MT had more muscle activity in the condition with the ergonomic stool compared to each one of the other conditions (*p* < 0.001) in the three tasks. Moreover, the difference among these three conditions also reached significance (*p* < 0.001).

**Table 2:** Mean of muscle activity in different ergonomic conditions and tasks by muscle.

Muscle	Task	CS Mean (SD)	ES Mean (SD)	ML Mean (SD)	ES and ML Mean (SD)	Significance		
						Wilks’ lambda	*F* Statistic	*p*-value
MT	TD	6.41 (3.65)	8.66 (4.91)	4.20 (2.42)	3.56 (2.00)	0.190	F_3.33_ = 46.77	<0.001
	TF	5.98 (3.35)	10.04 (5.66)	2.58 (1.47)	3.57 (2.00)	0.215	F_3.33_ = 40.14	<0.001
	TP	5.82 (3.27)	8.17 (4.60)	2.63 (1.54)	5.43 (3.06)	0.181	F_3.33_ = 49.83	<0.001
LES	TD	6.06 (4.25)	8.35 (5.86)	4.57 (3.08)	7.42 (5.16)	0.265	F_3.33_ = 30.52	<0.001
	TF	7.44 (5.20)	5.53 (3.69)	4.41 (2.99)	7.51 (5.22)	0.284	F_3.33_ = 27.66	<0.001
	TP	5.70 (4.01)	8.71 (6.12)	0.92 (0.62)	7.42 (5.12)	0.268	F_3.33_ = 30.04	<0.001

**Note:**

MT, medium trapezius; LES, lumbar erector spinae; TD, tooth drilling; TF, tooth filling; TP, tooth polishing; CS, conventional stool; ES, ergonomic stool; ML, magnification lenses.

**Figure 2:**
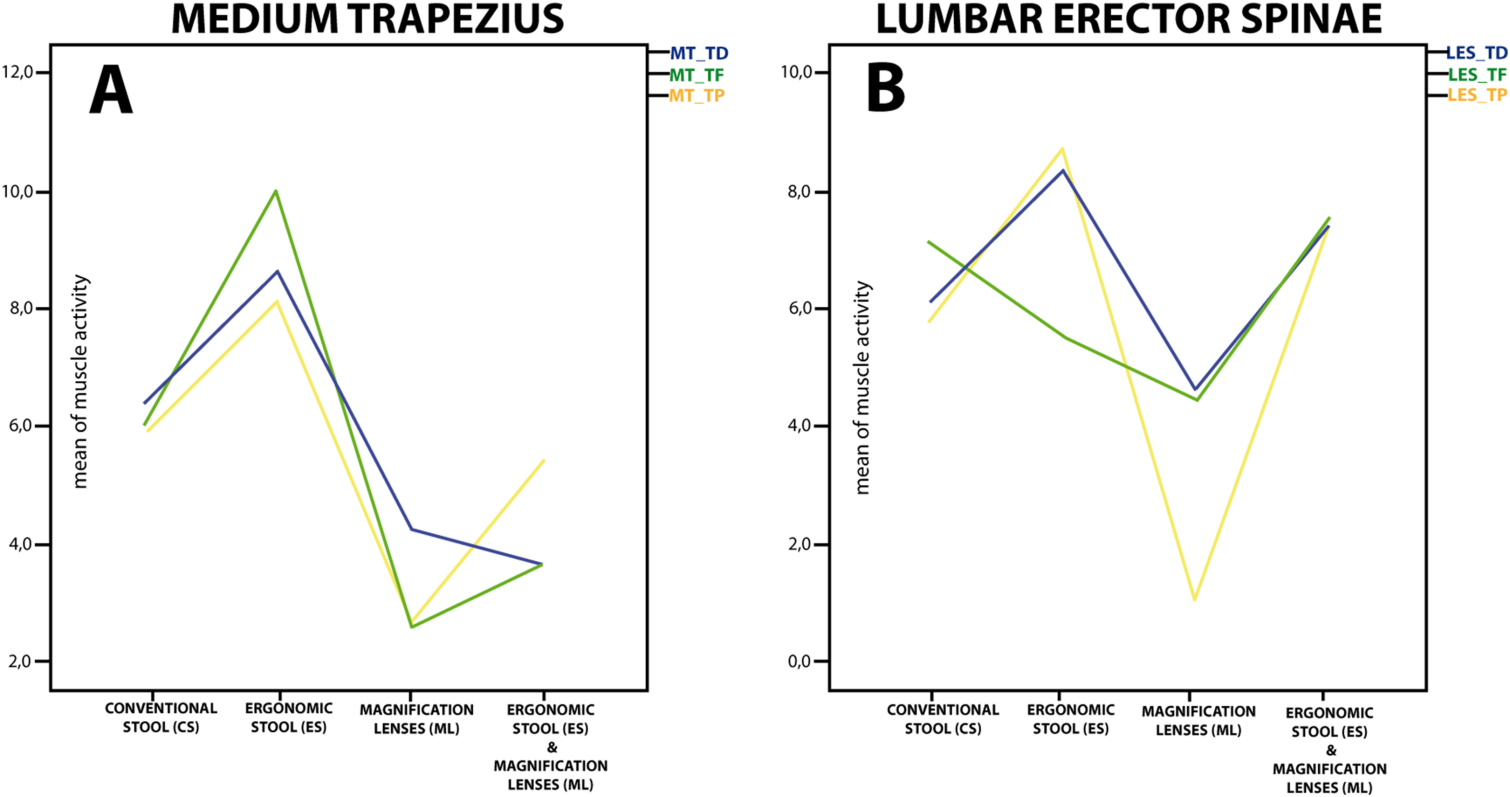
Mean of muscle activity for medium trapezius (A) and lumbar erector spinae (B) in different ergonomic conditions and tasks. MT, medium trapezius; LES, lumbar erector spinae; TD, tooth drilling; TF, tooth filling; TP, tooth polishing.

**Figure 3:**
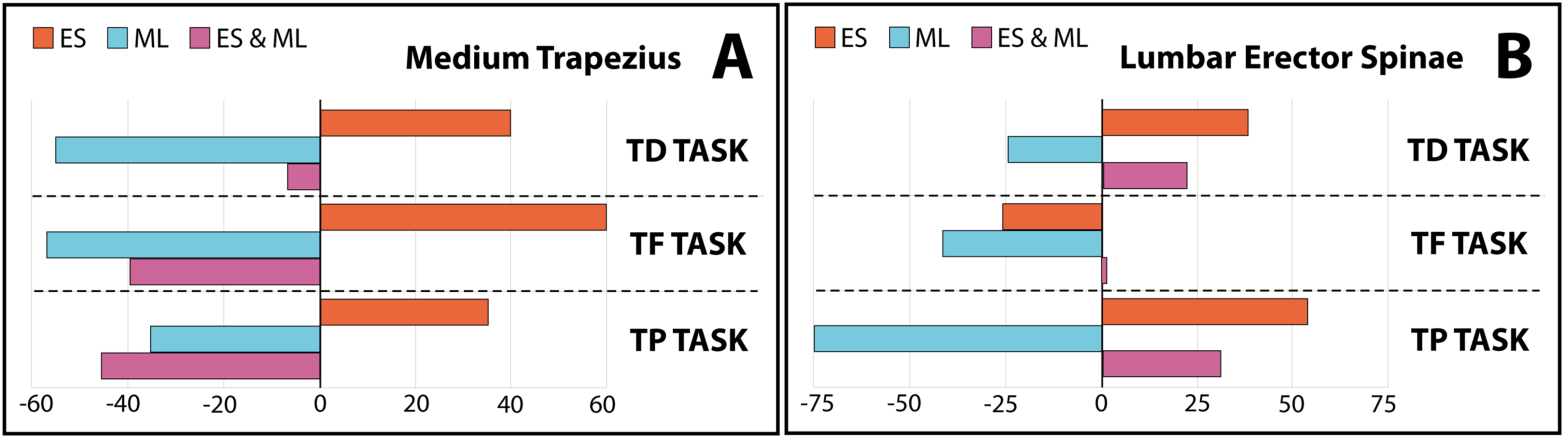
Percentage of change in muscle activity of (A) medium trapezius and (B) lumbar erector spine (reference: conventional stool) by ergonomic support. ES, ergonomic stool; ML, magnification lenses; ES & ML, ergonomic stool and magnification lenses; TD, tooth drilling; TF, tooth filling; TP, tooth polishing.

Figure 2A depicts the pattern of change between these three conditions (conventional stool, ergonomic stool, magnification lenses, and ergonomic stool and magnification lenses), which was similar for the three tasks. Moreover, this figure displays how the lowest muscle activity of MT occurred when participants used the magnification lenses. Figure 3A shows the percentage of change of the ergonomic supports regarding the conventional stool in order to quantify patterns of change. Additionally, for all three tasks, magnification lenses and the combination (the ergonomic stool and magnification lenses) reduced MT muscle activity in a range between 6.7% and 56.8%, while the isolated use of the ergonomic stool increased muscle activity.

Concerning the LES, we obtained a different main effect of the ergonomic conditions on muscle activity in the three tasks (Table 2). Bonferroni post hoc tests also showed differences between the three ergonomic supports (*p* < 0.001). However, the difference between the conventional stool and the ergonomic stool showed a different pattern regarding LES activation for the tooth filling task (Fig. 2B). The muscle activity of LES using the ergonomic stool was significantly higher than using the conventional stool alone for the tooth drilling and tooth polishing tasks (8.35 vs. 6.06 *(p* < 0.001) and 8.71 vs. 5.70 *(p* < 0.001), respectively). However, this muscle showed a significant decrease of activity during the tooth filling task (5.53 vs. 7.44). Furthermore, the muscle activity of the LES using magnification lenses was lower in all three tasks compared to using the conventional stool, ergonomic stool, and ergonomic stool and magnification lenses.

Figures 3A and 3B display and quantify all these patterns of change compared to the conventional stool. These figures also show how the magnification lenses were able to reduce LES muscle activity during the three tasks (between 24% and 74%).

## Discussion

This is the first study to evaluate the effect of different ergonomic supports on the muscle activity of the back muscles. Our findings show that magnification lenses are effective for decreasing the muscle activity of MT and LES during the three tasks of posterior composite restoration, compared to standard practice using the conventional stool. However, we observed a tendency for the opposite effect to occur with the use of the ergonomic stool or the combination of the same with magnification lenses (ergonomic stool and magnification lenses), by increasing the muscle activity in almost all tasks performed. This means that the increased muscle activity that occurs with the ergonomic stool is not modified by use of the magnification lenses, when used together with the stool in the case of the LES muscles.

Our sEMG results can only be partially compared with previous studies. In fact, few studies (Tran et al., 2016; De Bruyne et al., 2016) have evaluated the muscle activity of LES associated to the ergonomic stool. The ergonomic stool increases LES muscle activation during both the tooth drilling and tooth polishing tasks. However, these findings contradict those reported by other authors. A possible explanation for this could be that these authors used a different type of stool than that used in the study. While Tran et al. (2016) used a conventional chair incorporating a chest support, De Bruyne et al. (2016) used two different chairs, thus modifying hip flexion.

We agree with most authors who have found that the ergonomic stool generates a significantly decreased spinal flexion (Haddad et al., 2012; De Bruyne et al., 2016), improving dentists’ posture. However, the increase in muscle activation detected in our study supports the idea that optimal posture requires active maintenance of the physiological lumbar lordosis, and, therefore, higher levels of activation of LES and MT.

The use of magnification lenses produced the lowest levels of muscle activity in the MT. According to several authors, these prismatic lenses enable a better field of vision without the need to bend forward as much, leading to less strain. This may allow dentists to reduce neck flexion and adopt a more upright posture (Lindegård, Gustafsson & Hansson, 2012; Plessas & Bernardes Delgado, 2018). However, to date, no studies differentiate the posture generated by the magnification lenses and the ergonomic stool.

According to our results, the combination of the ergonomic stool and magnification lenses was similar with regards the muscle activity of MT compared to isolated use of magnification lenses in two of the three tasks. A possible reason for this could be a positive moderating effect on the posture from using the magnification lenses. In other words, the isolated use of the ergonomic stool generates an increase in muscle activation of MT, this effect is then mitigated by the combined action of magnification lenses. In our opinion, this surprising effect cannot be attributed to measurement errors because we found very reliable measures in our preliminary study (Table 1) with a percentage of standard error below 10%. Although we were unable to find reliability studies of the sEMG in this population, our results reveal that the sEMG is a reliable tool for measuring muscle activity of the back in dentists.

Regarding the muscle activity of the LES, a clear pattern of change was not observed between the different ergonomic supports. Only the use of magnification lenses produced a clear decrease in muscle activity. The effect of the ergonomic stool and the combination of the ergonomic stool and magnification lenses was higher compared to the conventional stool in two of the three tasks. These findings highlight the fact that the ergonomic stool requires the maintenance of lumbar lordosis, increasing the muscular activity of the LES during dental procedures.

This study has several strengths. First, our experimental protocol was similar to previous work, which facilitated comparison. Thus, participants used a simulated workstation with a phantom head model and were positioned in an ergonomic position with an ideal working distance; moreover, all prior studies allowed for rest periods between tasks to avoid exhaustion and used the MVIC to relativize the data obtained by the sEMG (Haddad et al., 2012; Tran et al., 2016; De Bruyne et al., 2016). Second, our sample size was greater than previous studies, such as Haddad et al. (2012), De Bruyne et al. (2016) and Tran et al. (2016) (using *n* < 30). Third, while these previous researchers used brief sEMG recording times (ranged from 20 to 30 s), we performed uninterrupted measurements of the entire task (180 s). In our opinion, these measures may be more representative of the reality of dentists’ daily work.

A limitation of the present study was that we did not collect the postural changes of dentists during tasks, as our only outcome measure was muscular activity. Another limitation was that the sEMG activity of MT and LES was only recording on the dominant side and no inference can therefore be made regarding the contraction pattern of muscles investigated on the non-dominant side.

## Conclusions

In conclusion, this study found that the ergonomic stool increases the muscle activity of MT and LES muscles to a greater degree compared to magnification lenses. The combination of the ergonomic stool and magnification lenses produces a different effect on the activity of both muscle groups. For the LES muscles, although the combination of supports leads to a similar muscle activation compared to the isolated use of the ergonomic stool (use of magnification lenses does not generate a change), in the MT muscle, the prevailing effect is that of the magnification lenses, as the ergonomic stool did not lead to changes.

The posture adopted by dentists may be different in these two ergonomic supports, therefore the use of these should not be generalized. A comprehensive analysis of the posture adopted by dentists in their working environment in needed to implement specific ergonomic measures. Future studies may want to expand the research to include other effects of ergonomic supports on muscle activity in different tasks, as well as possible interactions between the same.

## Supplemental Information

10.7717/peerj.8028/supp-1 Supplemental Information 1 Raw data.

## Additional Information and Declarations

### Clinical Trial Registration

The following information was supplied regarding Clinical Trial registration: The University of Murcia granted this study with Ref: 986/2014.

### Data Availability

The following information was supplied regarding data availability: The raw measurements are available in the Supplemental File.
